# Degrees of H2AX phosphorylation correlate with unique features of the intratumoral immune microenvironment in colorectal carcinomas

**DOI:** 10.1093/oncolo/oyag116

**Published:** 2026-03-30

**Authors:** Enrico Berrino, Sara Erika Bellomo, Maria Costanza Aquilano, Marta Falcinelli, Anita Chesta, Emanuele Valtorta, Daniela Zampieri, Gianluca Mauri, Giuseppe Sala, Silvia Marsoni, Alberto Bardelli, Andrea Sartore-Bianchi, Salvatore Siena, Anna Sapino, Emanuela Bonoldi, Fabrizio d’Adda di Fagagna, Caterina Marchiò

**Affiliations:** Pathology Unit, FPO-IRCCS Candiolo Cancer Institute, Candiolo, 10060 Italy; Department of Medical Sciences, University of Turin, Turin, 10124Italy; Pathology Unit, FPO-IRCCS Candiolo Cancer Institute, Candiolo, 10060 Italy; Department of Hematology, Oncology, and Molecular Medicine, Niguarda Cancer Center, Grande Ospedale Metropolitano Niguarda, Milan, 20162 Italy; IFOM ETS—The AIRC Institute of Molecular Oncology, Milan 20139, Italy; Pathology Unit, FPO-IRCCS Candiolo Cancer Institute, Candiolo, 10060 Italy; Department of Hematology, Oncology, and Molecular Medicine, Niguarda Cancer Center, Grande Ospedale Metropolitano Niguarda, Milan, 20162 Italy; Pathology Unit, FPO-IRCCS Candiolo Cancer Institute, Candiolo, 10060 Italy; Department of Hematology, Oncology, and Molecular Medicine, Niguarda Cancer Center, Grande Ospedale Metropolitano Niguarda, Milan, 20162 Italy; Department of Oncology and Hemato-Oncology, Università degli Studi di Milano (La Statale), Milan, 20122 Italy; Department of Hematology, Oncology, and Molecular Medicine, Niguarda Cancer Center, Grande Ospedale Metropolitano Niguarda, Milan, 20162 Italy; Department of Oncology and Hemato-Oncology, Università degli Studi di Milano (La Statale), Milan, 20122 Italy; IFOM ETS—The AIRC Institute of Molecular Oncology, Milan 20139, Italy; IFOM ETS—The AIRC Institute of Molecular Oncology, Milan 20139, Italy; Department of Oncology, Molecular Biotechnology Center, University of Torino, Torino 10126, Italy; Department of Hematology, Oncology, and Molecular Medicine, Niguarda Cancer Center, Grande Ospedale Metropolitano Niguarda, Milan, 20162 Italy; Department of Oncology and Hemato-Oncology, Università degli Studi di Milano (La Statale), Milan, 20122 Italy; Department of Hematology, Oncology, and Molecular Medicine, Niguarda Cancer Center, Grande Ospedale Metropolitano Niguarda, Milan, 20162 Italy; Department of Oncology and Hemato-Oncology, Università degli Studi di Milano (La Statale), Milan, 20122 Italy; Pathology Unit, FPO-IRCCS Candiolo Cancer Institute, Candiolo, 10060 Italy; Department of Medical Sciences, University of Turin, Turin, 10124Italy; Department of Hematology, Oncology, and Molecular Medicine, Niguarda Cancer Center, Grande Ospedale Metropolitano Niguarda, Milan, 20162 Italy; IFOM ETS—The AIRC Institute of Molecular Oncology, Milan 20139, Italy; IGM and Institute of Molecular Genetics—CNR (National Research Council), Pavia, 27100 Italy; Pathology Unit, FPO-IRCCS Candiolo Cancer Institute, Candiolo, 10060 Italy; Department of Medical Sciences, University of Turin, Turin, 10124Italy

**Keywords:** yH2Ax, MMR deficiently, MMR proficiency, colorectal cancer, immune infiltration

## Abstract

**Background:**

The phosphorylated form of the histone H2AX (γH2AX), a sensor of DNA double-strand breaks (DSB), can serve as a biomarker of DNA damage and therapy response. This study aimed to evaluate the association between γH2AX expression and pathological, molecular, and immune features in colorectal cancer (CRC).

**Patients and methods:**

Levels of γH2AX were assessed by immunohistochemistry in a cohort of 198 CRCs, alongside immune-related markers (CD3 and CD8). A sub-cohort of 65 CRCs (26 γH2AX^-^ and 39 γH2AX^+^) underwent RNA extraction and gene expression profiling using the IO360 Nanostring Panel to infer immune cell composition. Overall survival data were analyzed for exploratory correlations.

**Results:**

γH2AX^+^ CRCs (155/198, 78%) were significantly associated with higher stage and tumor grade (*P* < .01). A lower γH2AX prevalence was found in MMR-deficient tumors (64%) compared to MMR-proficient cases (81%, *P* = .05). γH2AX^+^ tumors showed increased CD3^+^ cell infiltration in the overall population (*P* = .038) and in MMR-proficient CRCs (*P* = .028). Gene expression analysis revealed higher T-cell counts (*P* < .01) and reduced B-cell abundance (*P* < .01) in γH2AX^+^ CRCs. Unsupervised clustering identified 3 immune subgroups with differential γH2AX accumulation. Cluster #3, enriched in γH2AX^+^ tumors, displayed increased CD8^+^ T-cells and conferred the best survival outcome.

**Conclusion:**

Elevated γH2AX expression correlates with MMR proficiency, aggressive histopathologic features, and a distinctive immune-active microenvironment in CRC. These findings may support γH2AX as a marker of immune-modulated CRC subgroups with potential prognostic and therapeutic relevance.

Implications for Practice Our findings identify γH2AX as a novel biomarker defining a distinct subgroup of mismatch repair–proficient colorectal cancers (MMRp CRCs) with molecular and immune features resembling MMR-deficient/MSI tumors. MMRp/γH2AX^+^ lesions exhibit increased CD3^+^ and CD8^+^ T-cell infiltration and improved survival, suggesting a favorable immune-active phenotype. Incorporating γH2AX evaluation into diagnostic workflows may enhance prognostic accuracy, refine patient stratification, and support personalized treatment approaches. γH2AX may contribute to refining the immunobiological stratification of MSS CRCs thus broadening the potential applicability of immune-based therapies beyond MSI tumors.

## Introduction

Genomic instability is essential for cancer initiation^1^ and represents a major vulnerability.^2^^,^^3^ Several pharmacological treatments, from chemotherapy to PARP inhibitors, have been developed to exploit these genetic susceptibilities for cancer treatment.^4–8^ Several oncogenic events are associated with DNA damage and the concurrent activation of the DNA Damage Response (DDR) machinery.^9–12^ Among these DNA injuries, Double-Strand Breaks (DSBs), which are induced by genotoxic agents^13^ and ionizing radiation (IR),^14^ are repaired by homologous recombination (HR) or by non-homologous end joining (NHEJ) mechanisms.^15–17^ Upon DSB generation, the DDR protein kinases activation lead to the phosphorylation of the histone H2AX [phosphorylated(y)-H2AX] which spread across kilobases from the break.^18^^,^^19^ yH2AX accumulation, organized in nuclear foci,^20^ has been detected in several pre-malignant and malignant lesions,^21–25^ and recent findings defined a pivotal role in prevention of carcinogenesis.^26^ In colorectal cancer (CRC) DSB accumulation is detrimental, however genomic instability represents a therapeutic vulnerability largely unexplored.^27^ Studies evaluating the yH2AX distribution in CRC are scarce. The largest study so far includes 92 CRCs stained by immunohistochemistry (IHC) and demonstrating that the higher the accumulation the more advanced stage and worse clinical outcome.^28^ However, poor correlations could be performed due to the absence of the mismatch repair (MMR) status, a major carcinogenetic driver in CRC. MMR deficiency (MMRd) leads to peculiar phenotypical features, able to modulate both cancer cells and tumor microenvironment.^29^ MMRd tumors usually show meaningful immune infiltration, especially by T-cells, due to an elevated repertoire of neoantigens associated to high tumor mutational burden.^30–32^ In this scenario, less is known about yH2AX accumulation and the immune infiltrate. A mild correlation of yH2AX with CD3 and CD4 expression was reported in early-stage lung cancers,^33^ and similarly a slight but not significant correlation with TILs was detected in 76 metaplastic breast carcinomas.^34^

Here, we report on yH2AX accumulation in a sequential cohort of 200 CRCs, evaluating its relationship with several pathological features, with a focus on MMR status and immune microenvironment.

## Material and methods

### Patient cohort

We collected an unselected sequential cohort of therapy-naïve 200 CRCs (2018—2020) at Grande Ospedale Metropolitano Niguarda, Milan, Italy, within the frame of the AlfaOmega Master Observational Trial (Ethical Committee of Grande Ospedale Metropolitano Niguarda, #145-07042020; NCT04120935 and NCT05101382).^35^ This study was conducted in accordance with the Declaration of Helsinki and the International Conference on Harmonisation and Good Clinical Practice guidelines.

The following clinical data were recorded: sex, age at diagnosis, type of treatment, stage, side, *RAS/BRAF* status, occurrence of relapse, and last follow-up or death. Full histopathological parameters were recorded, including results for MSH2/MSH6/MLH1/PMS2 expression by IHC.^3^

### Immunohistochemistry

**Figure 1.**
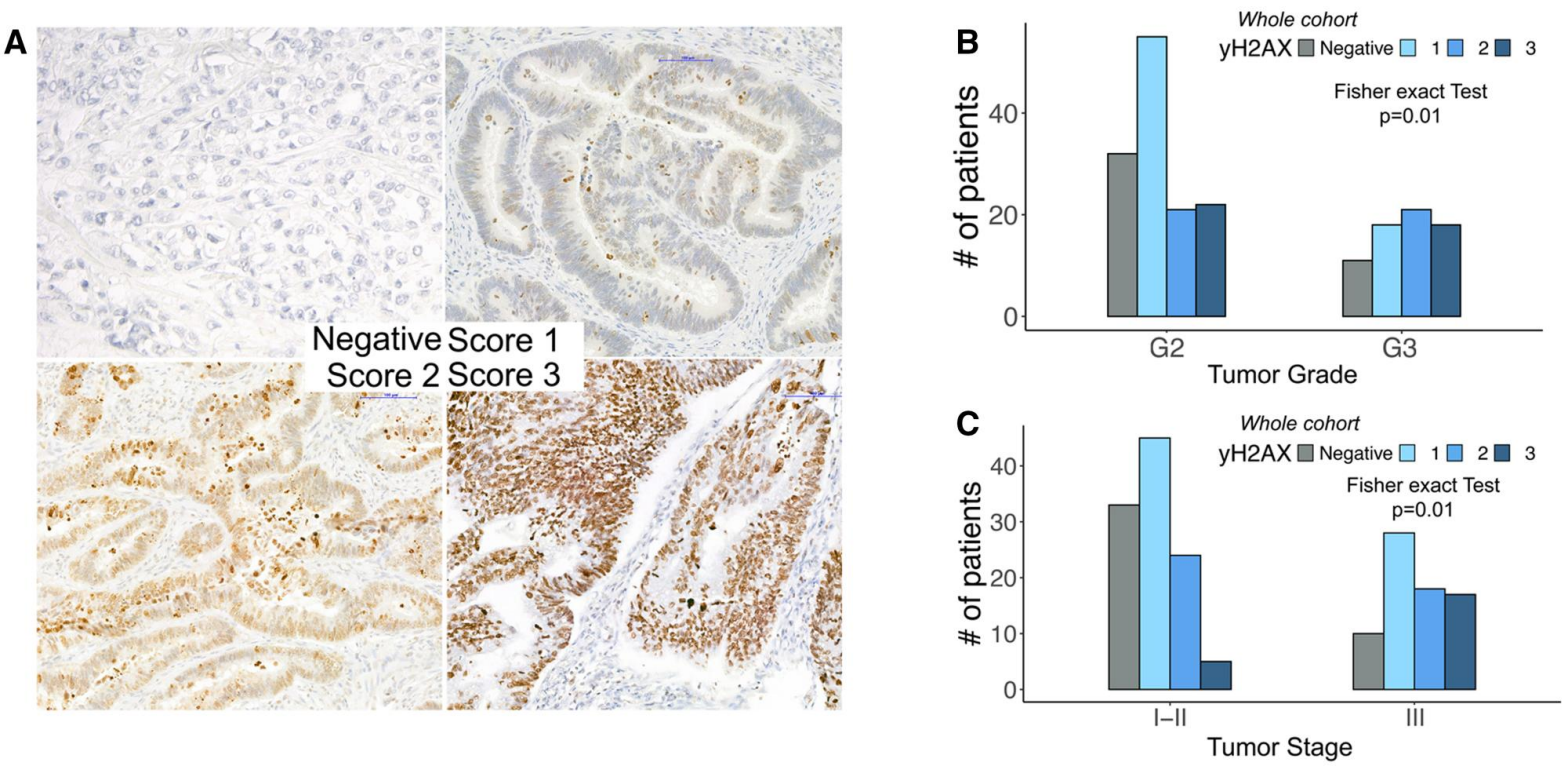
yH2AX accumulation in the CRC cohort. (A) Example of increasing yH2AX staining in CRCs, from negative to score 3. (B) Barplot and contingency table of yH2AX score in differentiated (G2) vs dedifferentiated G3 CRCs revealed a differential yH2AX accumulation in respect to the tumor grade. (C) Barplot and contingency table of yH2AX score in earlier (stage I-II) vs advanced (stage III-) G3 CRCs revealed a differential yH2AX accumulation in respect to the tumor staging.

Five micron-thick formalin fixed paraffin embedded (FFPE) sections were stained with the anti-gamma H2A.X antibody (phospho S139, 1:1000 diluted, ab11174) on the Dako Omnis instrument (Dako-Agilent, USA). Two pathologists assessed the accumulation of yH2AX reporting both nuclear intensity of staining and percentage of positive cells into a 4-tier scoring system, from 0 (absent staining) to 3 (strong staining) (Figure 1A).

Samples were also tested for CD3 (Polyclonal, Ready-to-Use, Dako-Agilent) and CD8 expression (Clone C8/144B, Ready-to-Use, Dako-Agilent) on the Dako Omnis instrument (Dako-Agilent). These markers were used to quantify the tumor infiltrating lymphocytes (TILs), including geographic distribution of the markers and the overall expression within the tumor area.

### RNA extraction

RNA-based analyses were performed on 85 CRCs selected for adequate tumor content, optimal RNA quality, and representativeness of γH2AX expression. Three 8-μm sections were mesodissected for RNA extraction using the Maxwell FFPE RSC kit (Promega, USA). RNA was quantified by Qubit (Thermo Fisher Scientific, USA), and analyzed for integrity and fragment distribution using the High Sensitivity RNA ScreenTape assay (RIN and DV200 metrics) on the Agilent 4150 TapeStation (Agilent, USA).

### Gene expression analysis

The gene expression of 770 genes was assayed by the PanCancer IO 360 panel, a targeted gene expression profiling (GEP) approach (NanoString Technologies, USA). Briefly, 500 ng of RNA were subjected to an overnight incubation at 56 °C (for 14 hours) with pre-designed oligo probes to capture the targeted genes. Exploiting the NanoString nPrep Station we purified samples, and the NanoString nCounter instrument read the associated fluorescence (NanoString Technologies). Raw results were elaborated using the nSolver Software (NanoString Technologies), exploiting the nSolver Advanced Analyses package.^35^ Twenty samples did not meet QC criteria, resulting in a final analytical cohort of 65 cases. Volcano plots for the differential gene expression (DGE) among the yH2AX groups were generated using the ggplot R package using *P* < .0001 and FoldChange (Fc) expression >1.5 as cutoffs for significance. Raw and relative cell type score as well as the pathways scores were used to cluster the 65 patients using the *k*-means algorithm implemented in the ComplexHeatmap R package (clustering method: “ward.D,” clustering distance: “Euclidean”). We selected the best *k* using the silhouette score calculated with the “cluster” R package (v.2.1.6).

### Statistical analysis

Statistical analyses were performed using R software (v.4.0.3). For the correlative analysis, yH2AX accumulation was used to stratify the entire cohort for the IHC Scores (0, 1, 2, 3) and for the IHC Value (Negative vs Positive). We used the chi-square test for correlative analyses between yH2AX groups and clinico-pathological features, the t-test was applied for distribution comparison of both the CD8^+^ and CD3^+^ IHC expression values (%) and the RNA-inferred scores (both pathway and cell type scores). For survival analyses, Overall Survival (OS, calculated as the time from diagnosis to death or last follow-up date) was evaluated by the Kaplan–Meier method and analyzed with the log-rank test and by univariate and multivariate Cox Proportional Hazard Model with the r packages *survival* and *finalfit*. *P*-values with false discovery rate correction (FDR) < 0.05 were considered as statistically significant.

## Results

### yH2Ax accumulation across CRCs and correlation with clinico-pathological features

We successfully assessed yH2Ax protein accumulation by IHC in 198/200 CRC. In 2 cases pre-analytical issues led to non-uniform staining throughout the sections (cases excluded from further evaluation).

Forty-three of 198 tumors (22%) were classified as score 0, whereas 73/198 (38%) showed low expression (score 1), 42/198 (21%) intermediate expression (score 2), and 37/198 (19%) strong accumulation (score 3) (Figure 1A). By the dichotomous classifier [grouping tumors as γH2AX-negative (score 0) vs positive (score 1–3)], 43/198 CRCs (22%) were γH2AX negative, while 155/198 (78%) showed detectable expression.

We then cross-correlated both IHC classifier and scores with all the main clinic-pathological features (Table S1, summarized in Table S2). We identified few associations between yH2AX accumulation and patient characteristics: neither the primary site (colon vs rectum) nor the tumor side (left vs right) were significantly associated with differential yH2AX accumulation (Table S3). However, contingency table with Fisher’s exact test showed that positivity for yH2AX was more pronounced in the advanced stage (III-IV) compared to the early CRCs (*P* = .03 for yH2AX Value and *P* < .01 for yH2AX Score, Figure 1B). Moreover, the accumulation of yH2AX was not related with histology (adenocarcinoma not otherwise specified vs mucinous carcinomas) or with the percentage of mucinous component. More differentiated tumors (G2) showed a higher, yet not statistically significant, proportion of γH2AX-negative cases than poorly differentiated tumors (G3) using the dichotomous classifier (25% vs 16%, *P* = .17; Table 1). The full ordinal IHC score (0–3) revealed a significant distribution difference across tumor grades (Figure 1B, *P* < .01; Table S3). yH2AX negative lesions showed a reduced tumor budding (Bd0-1:86%) compared to yH2AX positive CRCs, characterized by a 36% of tumors with Bd2-3 (*P* = .05, Table 1).

**Table 1.** Correlation between yH2Ax expression and clinico-pathological features.

yH2Ax value crosstabs (*n* = 198)				
		Negative	Positive	*P*
**Histology**	ADK	33	10	.4
	Mucinous ADK	127	28	
**Mucinous component (%)**	0	19	90	.25
	1-25	8	27	
	26-49	6	10	
	50-100	10	28	
**Tumor grade**	G2	32	98	.17
	G3	11	57	
**Vascular invasion**	Absent	35	116	.37
	Present	8	39	
**Lympho-vascular invasion**	Absent	30	87	.1
	Present	13	68	
**Tumor budding**	Bd0	5	13	.05
	Bd1	32	86	
	Bd2	2	20	
	Bd3	4	35	
**MMR status**	MMRd	10	18	.05
	MMRp	33	137	
**Primary site**	Colon	38	141	.61
	Rectum	5	14	
**Colon side**	Right	26	85	.7
	Left	12	52	
**Stage**	I-II	33	92	.03
	III	10	63	

Overall, γH2AX accumulation was broadly distributed across CRCs and associated with aggressive features (grade, stage, and budding), rather than location or histological subtype.

### Correlation between yH2AX accumulation, MMR status and IHC biomarkers of immune cell infiltration

**Figure 2.**
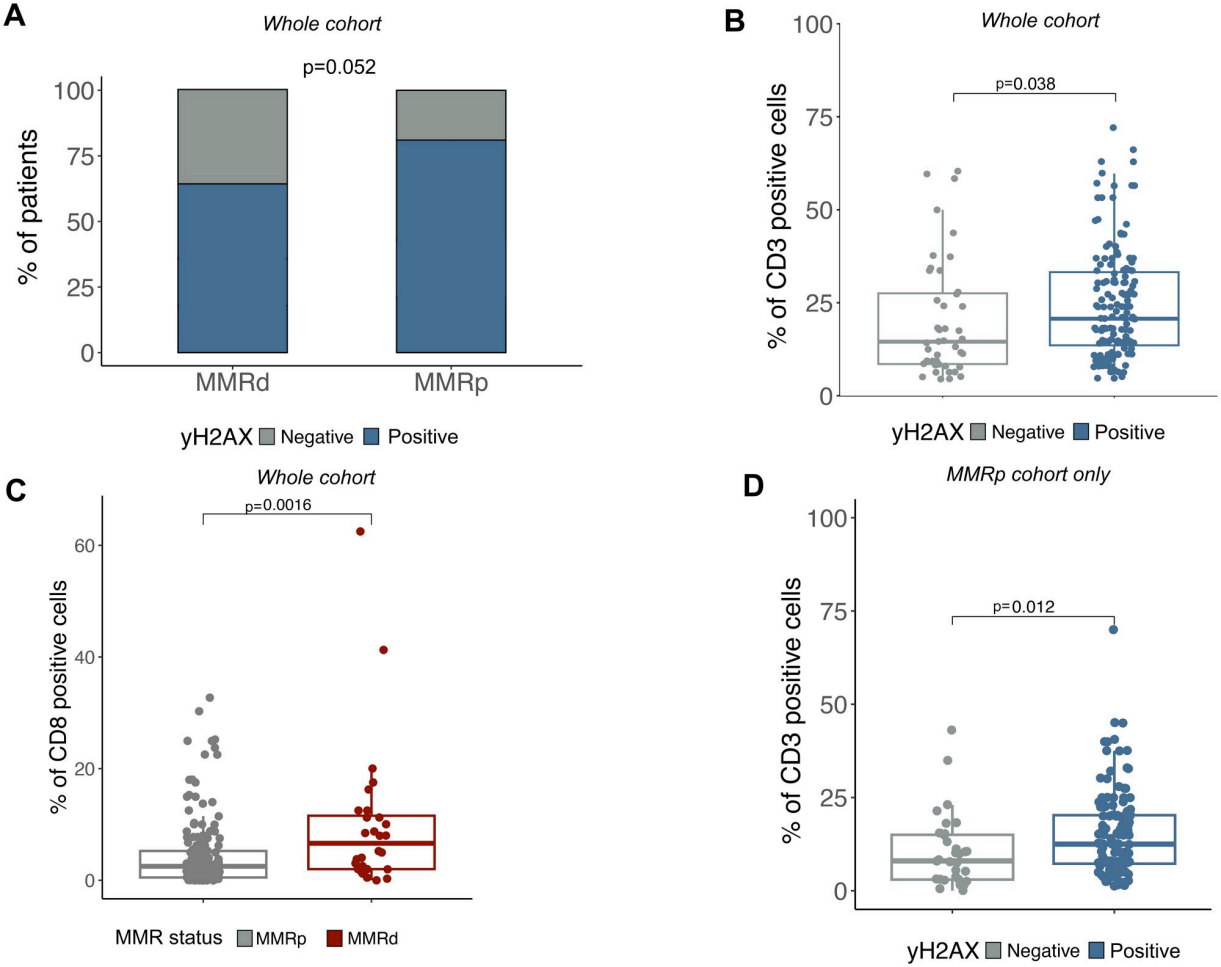
Correlation between yH2AX accumulation and CRC features. (A) Stacked barplot reporting the percentage of each yH2AX group and the MMR status. (B) Boxplot the CD3 positivity and the yH2AX score-based groups. (C) Boxplot the CD8 positivity and the MRR status-based groups. (D) Boxplot the CD3 positivity and the yH2AX value-based groups in the MMR proficient cohort.

Based on the assessment of MLH1/PMS2/MSH2/MSH6 expression our cohort was composed of 172/198 (86%) MMR proficient (MMRp) and 28 MMR deficient (MMRd) CRCs (14%). We investigated whether MMR status could be related with differential yH2AX accumulation. To avoid hyper-stratification of the small MMRd cohort (28 CRCs, distributed in 10 yH2AX negative, 8 with score 1, 5 with score 2 and 5 with score 3), we used the dichotomous separation into positive vs negative. We identified a lower prevalence of yH2AX positive CRC (18/28, 64%) in the MMRd lesions, compared to the MMRp CRCs (137/170, 81%, *P* = .05, Figure 2A and Table 1). All the MMRd CRCs were right-sided (26/28), thus precluding assessment of the correlation of the yH2AX staining related to side of primary tumor origin. No differences in terms of site (colon vs rectum) nor for colon tumor side (left vs right) were identified for the yH2AX accumulation in the MMRp cohort, in which the G2 CRCs confirmed to be less prone to the accumulation of this biomarker compared to poorly differentiated CRCs (G3 *P* = .02).

Subsequently, we wondered if immune infiltration could be associated with degrees of yH2AX accumulation. We started from a simple IHC-based approach assessing CD3- and CD8- positive T cells. In the 198 CRCs successfully stained with the yH2AX antibody a mean of CD3^+^ and CD8^+^ positivity of 14.5% and 5.3%, respectively, was observed. We then compared the distribution of immune markers within the yH2AX classes. A trend of reduced CD3^+^ staining was identified in the yH2AX negative CRCs, compared to the yH2AX positive CRCs [Negative: 6.0% (9.0-16.8), 1: 12.6% (12.3-17.8), 2: 13.0% (11.5-18.5), 3:12.5% (11.9-20.3)]. When using the IHC classifier (Negative vs Positive), we identified a significant increase of CD3^+^ cells in the yH2AX positive tumors (6.0 (9.0-16.8) vs 12.5 (13.4-17.2), *P* = .038, Figure 2B). No significant correlations between CD8 and yH2AX expression were observed (Table S4).

We next assessed the distribution of the immune markers in the context of the MMR status: beside a superimposable amount of CD3^+^ cells, MMRd tumors exhibited a significantly higher CD8^+^ cells compared to the MMRp CRCs (6.6% (4.9-15.2) vs 2.5% (3.6-5.6), *P* = .002, Figure 2C). This feature prompted us to consider the yHA2X-immune marker association separately for MMRd and the MMRp cases. In the MMRd population (*n* = 28), we were not able to confirm the increase of CD3^+^ cells in the yH2AX positive lesions (*P* = .11), and no differences in CD8 expression were identified (*P* = .6). Conversely, yH2AX-high, MMRp tumors showed a significantly higher CD3 expression (*P* = .01, Figure 2D), whereas statistically significance was not reached when analyzing CD8 (*P* = .4, Table S4).

Taken together, these data show that the association between γH2AX and T-cell infiltration is mainly observed in MMRp tumors, with increased CD3+ T-cell levels in γH2AX-positive cases.

### Gene-expression based immune cell count and pathway scoring in the different levels of yH2AX accumulation

**Figure 3.**
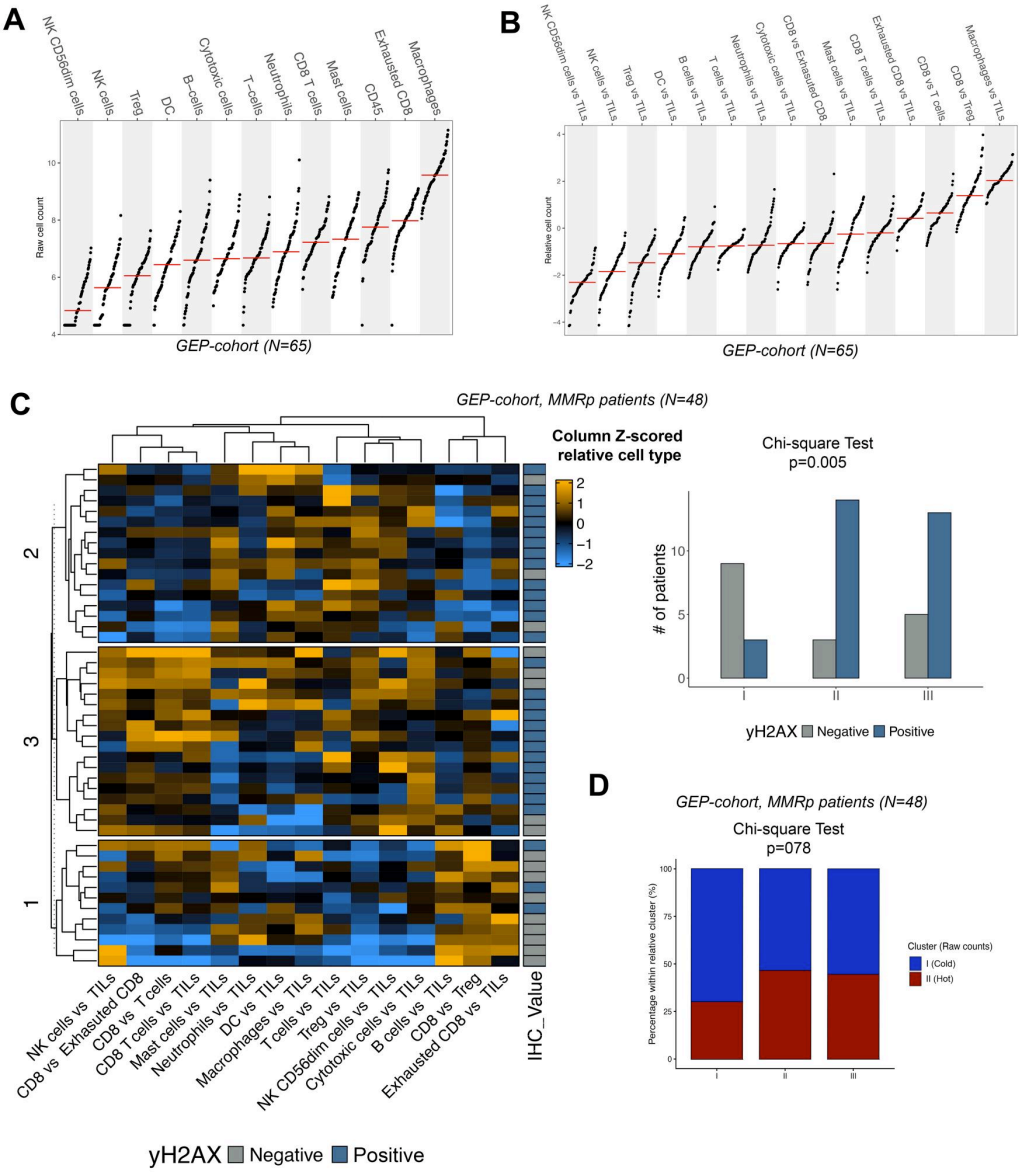
Gene-expression based immune cell type scores and the correlation with the yH2AX accumulation. (A) Distribution of the raw immune cell type count in the CRC cohort. (B) Distribution of the relative immune cell type count in the CRC cohort. (C) *K*-mean clustering of relative immune cell count in the MMR proficient CRC cohort, annotated for the yH2AX value with the barplot reporting the differential distribution of yHA2X accumulation within the *k*-mean identified clusters. (D) Stacked barplot reporting the differential distribution of raw immune cell type clusters (“hot” and “cold”) within relative immune cell type clusters.

Since the simple CD3/CD8 IHC assay revealed a significant correlation of different levels of yH2AX accumulation with the amount of CD3^+^ positive cell (a T cell universal biomarker), but no differences in terms of CD8^+^ positive cell distribution were observed, we aimed at exploring the composition of the immune infiltrate of CRCs. We exploited a GEP targeted approach on 65 representative samples (26 negative and 39 positive for γH2AX) in terms of MMR features and CD3/CD8 expression (summarized in Table S5). In addition to the immune cell inference scores, we assessed CD3 complex transcripts (*CD3D, CD3E, CD3G*) from the NanoString panel. *CD3D* and *CD3E* were significantly upregulated in γH2AX-positive tumors (*P* = .022 and *P* = .001), while *CD3G* showed no difference (*P* = .76). These results are consistent with CD3+ IHC and support increased T-cell abundance in γH2AX-positive CRCs (Figure S1). We inferred the immune cell count from transcriptomic data, generating for each patient a score defining the quantity of each immune cell type (raw count) and a score representing the ratio between each single cell type and the inferred total TILs (relative count). The distribution of the raw and relative counts is reported in Figure 3A-B. First, when we compared the distribution of raw and relative counts, yH2AX positive CRCs showed a higher count of relative T-Cells (Table 2, *P* < .01), in contrast with a lower abundance for relative B-Cells (Table 2, *P* < .01) compared to the yH2AX negative tumors. No other correlations were observed.

**Table 2.** Correlation yH2Ax expression and CD3 and CD8 positivity in the RNA-analysed cohort.

Cohort	yH2Ax negative vs positive	Wicox-test (*P*-value)
**All (*n* = 65)**	B cells/TILs*	.01
	T cells/TILs*	<.01
**MMRd only (*n* = 18)**	B cells/Tal*	.3
	T cells/TILs*	.07
**MMRp only (*n* = 47)**	B cells/TILs*	.01
	T cells/TILs*	.01

Therefore, we asked if a composite signature rather than a cell type score could be more relevant to dissect potential correlations with yH2Ax accumulation. To this end, we applied unsupervised clustering on both raw and relative cell count on the entire cohort. In terms of raw count, k-mean clustering (optimal k = 2, Figure S2A-B) stratified tumors for the quantity of immune infiltration, identifying a group of “hot” CRCs (Cluster II), enriched for all immune cell types, and mainly correlated with the MMR status (16/18, 89% belong to the cluster II, *P* < .01, Figure S2C). No associations between the yH2AX status and these clusters were identified, considering both the entire cohort and the CRCs stratified according to the MMR status (Figure S2D-F).

We next explored the ability of the relative cell count to group the CRCs. By this analysis we identified 3 groups with a trend of enrichment for yH2AX accumulation in cluster #2 and #3 (*P* = .12, Figure S3A-C). When we applied the same approach to MMRd and MMRp CRCs separately, the latter cohort reached a significant stratification of yH2AX accumulation (best k = 3, *P* = .02, Figure 3C) in contrast with the k-mean clustering for the MMRd cohort (best k = 2, *P* = .32; Figure S3D).

Figure 3C reports the clusters for the MMRp cohort: cluster #1 was mainly enriched of yH2AX negative lesions (9/13, 70%), whereas the other 2 clusters, with a different pattern of relative cell type composition, showed a larger number of yH2AX positive CRCs (14/17, 82% and 12/18, 67% for cluster#2 and 3, respectively). The relative-score clusters did not overlap with the hot/cold classification based on raw counts, with no association in MMRp tumors (χ^2^  *P* = .78; Figure 3D). Overall, while raw immune abundance reflected MMR status, relative composition better stratified γH2AX within MMRp CRCs.

**Figure 4.**
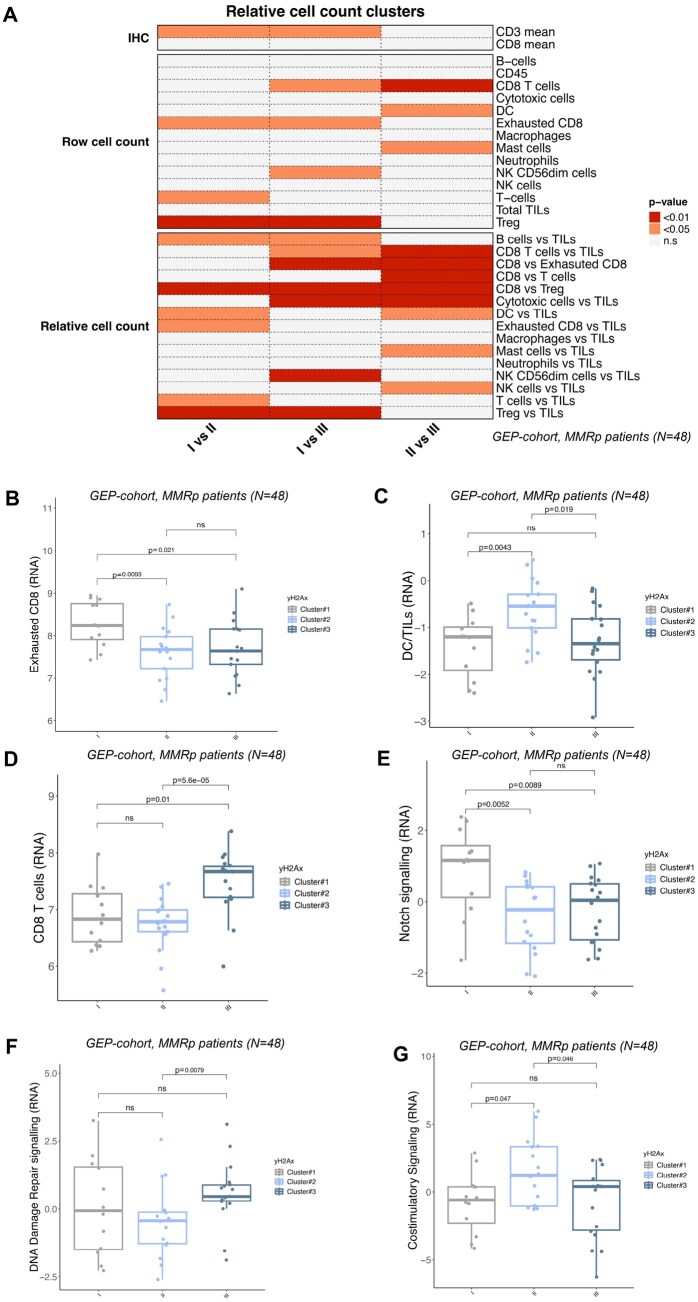
Correlation between the *k*-mean clusters, the immune microenvironment and the pathway activation inferred by the gene expression profiling. (A) Heatmap of the *P*-value reporting the significant comparisons between the immune clusters. (B) Boxplot of exhausted CD8 distribution within the immune clusters. (C) Boxplot of dendritic cell (DC)/TILs distribution within the immune clusters. (D) Boxplot of CD8 distribution within the immune clusters. (E) Boxplot of Notch signaling score within the immune clusters. (F) Boxplot of DNA damage repair signaling score within the immune clusters. (G) Boxplot of costimulatory signaling score within the immune clusters.

Next, we applied distribution test to characterize the IHC based score, the raw and the relative cell count within the 3 clusters. The *P*-value heatmap reports the significant results in Figure 4A. Cluster #1 showed a reduced expression of CD3 marker compared to the other 2 groups (*P* < .05 for both). In the same cluster, we observed a B-cell enrichment, for both raw and relative counts, in line with the yH2AX negative predominant composition. The same group was enriched also in exhausted CD8 T (Figure 4B) but depleted in regulatory T cells (Treg). Cluster #2 was characterized by a higher infiltration of dendritic cells (DC, both raw and relative), and by a higher relative quantity of T-cells/total TILs (Figure 4C). Finally, cluster#3 showed a clear increase in CD8 T-cells in the RNA-based test, for both the total and all the relative comparisons (vs TILs and vs all the other T cell types Figure 4D).

Through a differential expression analysis on the 770 analyzed genes, we identified few genes differentially expressed between cluster#2 and #3, whereas several genes varied between cluster#1 and the others (Figure S4). Finally, through the pathway score assessment, we evaluated the most differentially activated signaling pathway within the clusters. Although an unsupervised analysis did not identify combined signaling signatures able to reconstruct the immune-clusters, distribution test showed some pathways specific for each subgroup. As reported in Figure 4E, cluster#1 was clearly enriched for Notch activation compared to the 2 yH2AX positive clusters. Cluster #3 displayed a higher activation of the DNA-damage repair pathway, cluster#2 was characterized by a significant enrichment of costimulatory signaling (Figure 4F-G). Figure S5 reports the DDR genes differentially expressed between the 2 clusters: cluster#3 was enriched for *BRCA1/2*, *RAD51C*, *FANCA*, and *POLD1.*

Collectively, these findings demonstrate that γH2AX-positive MMRp tumors segregate into biologically distinct immune configurations, characterized by either priming/costimulatory or cytotoxic/DDR-associated axes.

### Explorative correlations between yH2AX accumulation and clinical outcomes

We collected OS data for all the patients; we excluded 19 patients since immediately metastasized after surgery. By applying an explorative Univariate Cox Proportional Hazard Model, adjuvant therapy emerged as the major stratifier of the cohort (HR: 0.29, range: 0.14-0.57, *P* < .001). Multivariate analysis (Table S6, Figure S6) reported a poor prognostic role for higher tumor grade [HR: 1.86 (1.00-3.47), *P* = .049], non-rectal localization (HR: 0.26, range: 0.08-0.80, *P* = .019) and a trend of lower OS for MSS lesions (HR: 2.35, range: 0.96-5.73, *P* = .061). In this scenario, yHA2X accumulation did not stratify the cohort for the survival, with forest plot (Figure S6) and Kaplan Meier plot (Figure S7) showing a non-significant, highly variable HR of 1.45 for yH2AX-positive CRCs. When focusing on MMRp lesions, the multivariate analysis returned significance only for tumor grade (HR: 2.59, range 1.48-4.54, *P* = .001), with adjuvant treatment not associated with a differential OS (Table S7).

**Figure 5.**
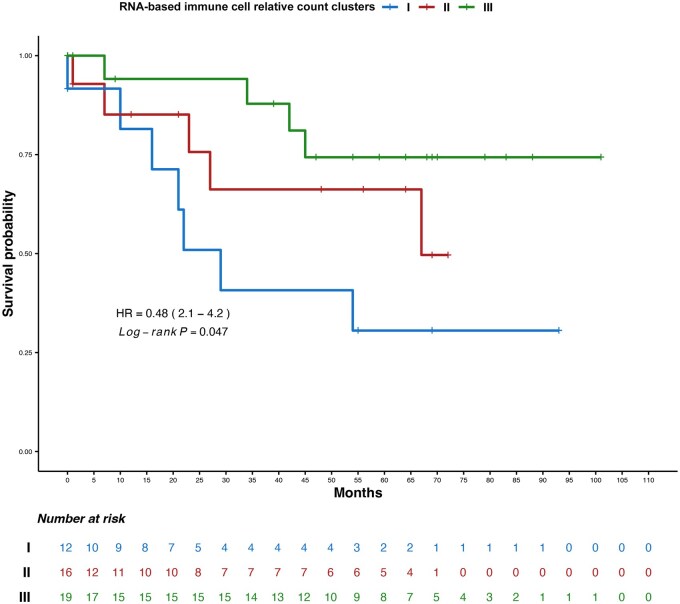
Overall survival Kaplan–Meier curves with HR and *P*-value for the relative cell count immune related clusters in the MMR proficient cohort analyzed for the gene expression profiling.

Next, we tested if the clusters based on the relative immune infiltration related to the yH2AX accumulation identified in the MMRp lesions was associated with OS. Multivariate analysis showed that cluster #3 (yH2AX positive) represented a clear-cut favorable factor compared to cluster #1, mainly composed by yH2AX negative (Table S8 and Figure S8). Kaplan-Meier (Figure 5) confirmed this trend, with cluster #2 showing an intermediate OS.

Overall, γH2AX accumulation alone did not significantly stratify survival; however, immune-based clustering within MMRp tumors identified subgroups with distinct prognostic trajectories.

## Discussion

In the era of the preclinical models several studies have reported on the exciting role of DNA damage and repair, defining several new predictive or prognostic cancer biomarkers.^36–38^ However, their validation often failed upon translation to real-world cancer cohorts.^39^^,^^40^ Here, we assessed yH2AX accumulation in a consecutive cohort of early CRCs. yHA2X positive lesions exhibited features of aggressiveness (higher tumor grade and stage) and yHA2X lack of expression was more frequently associated with MMRd lesions. An RNA-based classification of the immune-infiltration identified clusters polarized for yH2AX accumulation, mainly associated with specific relative and qualitative TILs content and differential clinical behavuiour.

Two major topics emerge from the yH2AX staining results across different cancer types. First, the discrepancy of the scoring system across different reports affects a reliable comparison.^21^^,^^28^^,^^41–44^ We recorded intensity and pervasiveness of the yH2AX staining, thus allowing a *post-hoc* binarization of data. Conversely, most of the published results did not consider degrees of expression, usually comparing pathological data between positive (high expressors) and negative (not high expressors) tumors.^21^^,^^41^^,^^43^^,^^44^ This scenario influences comparisons in terms of prevalence of yHA2X positivity across studies. Breast tumors showed a 30% positivity rate for yH2AX,^45^ more prevalent in triple negative lesions.^46^ A quarter of the early-stage lung carcinomas^21^ showed high yH2AX staining, whereas all the gastric cancers analyzed by Guo et al. showed a detectable level of staining.^43^ In a study focused on 92, all stages CRCs, Lee and colleagues developed a 3-level system, in which the yH2AX grade II and III, representing the positive lesions, led to a 61% positivity rate. Although slightly lower, this prevalence is in line with our data (positivity rate of 78%): indeed, grade I by Lee and colleagues (negative for yH2AX) also contained lesions with weak accumulation, thus explaining this minimal misalignment.^28^

In our study we correlated yH2AX expression levels to pathological features routinely recorded in clinical practice that help prognostically stratify CRC patients. Less differentiated lesions showed higher yH2AX expression levels and yH2AX accumulation correlated with tumor budding: about 90% of Bd2 and Bd3 CRCs expressed yH2AX, whereas 40% of Bd0 and Bd1 lesions lacked yH2AX expression. The only report assessing tumor budding is related to cervical cancer and confirmed a higher yH2AX accumulation in the cells at the invasive front of the tumor, suggesting a potential role for yHA2X as sensor for tumor invasion.^44^ Furthermore, in our cohort MMRd lesions showed a higher prevalence of yHA2X negative tumors (36% vs 18% of MMRp). The other CRC study on record did not assess this feature, and no data on a potential relationship between loss of MMR and the level of phosphorylation of yHA2X are on record.^47^^,^^48^

The second part of the study was triggered by 2 assumptions: (1) CRCs are conceptually classified as “hot” or “cold” by the extent of the infiltrating immune-cell microenvironment and the response to immune-therapies;^49^^,^^50^ (2) in “hot” lesions the recruitment of the immune cells is generally triggered by neoantigens produced by an accumulation of DNA alterations not repaired in a MMR deficiency scenario. A recent review reported a correlation between DSBs and the inception of an immune-fertile tumor infiltrate;^27^ hence, we wondered if a marker of DBSs (yH2AX) was associated to a specific immune cell type microenvironment.

Since MMRd tumors, less positive and usually immune-inflamed, represented a potential confounder for stratification, we focused on MMRp lesions, and we observed that relative quality rather than the quantity clustered and correlated with yH2AX accumulation. Recently, MSS lesions have been distinguished in 2 immune-related groups: the type II, like MSI lesions for the immune infiltration, characterized by increased CD8+ and Tregs, and the type I, characterized by a large extend of M2 macrophages and a higher proportion of B-cells.^51^

In our hands, the relative amount of each immune cell-type inferred by RNA profiling identified 3 groups. Cluster#1, mainly composed by MMRp/yH2AX^-^ lesions, characterized by an increase of B-cells and an enrichment of exhausted CD8^+^ T cells, a hallmark of cancer immune evasion in MMRp CRCs.^52^ Clusters #2 and #3, enriched in yHA2X^+^ CRCs, differed from each other for CD8+ T-cells, and both showed a greater raw and relative count of Tregs compared to the Cluster #1. In this scenario, although higher amount of CD8+ T-cells are proper of MSI favorable CRCs,^53^ recent single-cell analysis demonstrated that MSS/CD8^+^ T-cell high lesions show a low diversity of the TCR, leading to a group of CRCs less “immunogenic” then expected.^54^

Importantly, the 2 γH2AX-positive MMRp clusters were not redundant but reflected distinct immune configurations: Cluster #2 was enriched in dendritic cells and costimulatory signalling, consistent with an antigen-presentation/priming-oriented milieu. This suggests an immune context driven by antigen processing and T-cell priming rather than effector dominance. In contrast, cluster #3 showed a stronger CD8+ T-cell component with a prominent DDR program, indicating a more effector-oriented landscape linked to sustained DNA damage signalling. Notably, in MSS CRC, CD8-high states may also reflect bystander (“pseudo-hot”) activation rather than fully tumor-reactive immunity. Therefore, while both clusters are γH2AX-positive, cluster #2 appears to define a priming/costimulatory axis of immune engagement, whereas cluster #3 reflects a cytotoxic/DDR-associated axis, underscoring the heterogeneity of γH2AX-positive MMRp tumors.^55–57^

Finally, we exploratory evaluated yH2AX as a prognostic biomarker. In the entire population multivariate analysis revealed a 1.5-fold (but not significant) HR for yH2AX-positive lesions, which confirms a previously reported trend in a mixed cohort of early and advanced CRCs.^28^ A different scenario was encountered considering the yH2AX-immune related clusters: the yH2AX-negative group showed the worst survival, whereas cluster III (yH2AX^+^, CD8+ and Treg high) displayed the best outcome overall, with a 0.17-fold HR compared to cluster I. Although preliminary, this observation may suggest that a subset of γH2AX-positive MMRp tumors shares selected immune and clinical features with MSI/MMRd CRC; however, the heterogeneity observed within γH2AX-positive cases indicates that additional immune-contexture characteristics contribute to this stratification.

The main limitation of the study is related to the retrospective nature of the cohort, affecting both the choice of a targeted gene expression profiling method and the clinical data collection. The archival samples available led to perform transcriptomic analyses on a technically feasible subset rather than the entire cohort. Nevertheless, the analyzed subset remained highly representative of the overall cohort in terms of clinico-pathological characteristics and γH2AX distribution.

Finally, when considering survival data, the prognostic role of our biomarkers could have been influenced by the heterogeneity in patient management.

In conclusion, the assessment of γH2AX accumulation, as a proxy of DSBs, identifies a subset of MSS CRCs characterized by distinct immune configurations. Within γH2AX-positive MMRp tumors, heterogeneous immune patterns emerge, including a subgroup displaying immune and clinical features partially overlapping with those observed in MSI/MMRd CRC. γH2AX may contribute to refine the immunobiological stratification of MSS CRCs rather than serving as a standalone surrogate of MSI-like behavior. Further clinical validation in homogeneously treated cohorts is warranted.

## Supplementary Material

oyag116_Supplementary_Data

## Data Availability

This study includes no data deposited in external repositories. Source data and Extended Data files are provided with this paper. All other data supporting the findings of this study are available from the corresponding author on reasonable request.

## Submission declaration and verification

The present manuscript was not previously published or under consideration elsewhere. We presented these preliminary results as poster at the 2025 USCAP congress (DOI: 10.1016/j.labinv.2024.102794)
