# Supplementary material for: Degrees of H2AX phosphorylation correlate with unique features of the intratumoral immune microenvironment in colorectal carcinomas
Source: Oncologist. 2026 Mar 30;31(5):oyag116. doi: 10.1093/oncolo/oyag116 (PMC13071407; doi:10.1093/oncolo/oyag116)
Supplement: oyag116_Supplementary_Data [file oyag116_supplementary_data.zip › Supplementary Figure 8_rev1.pdf]

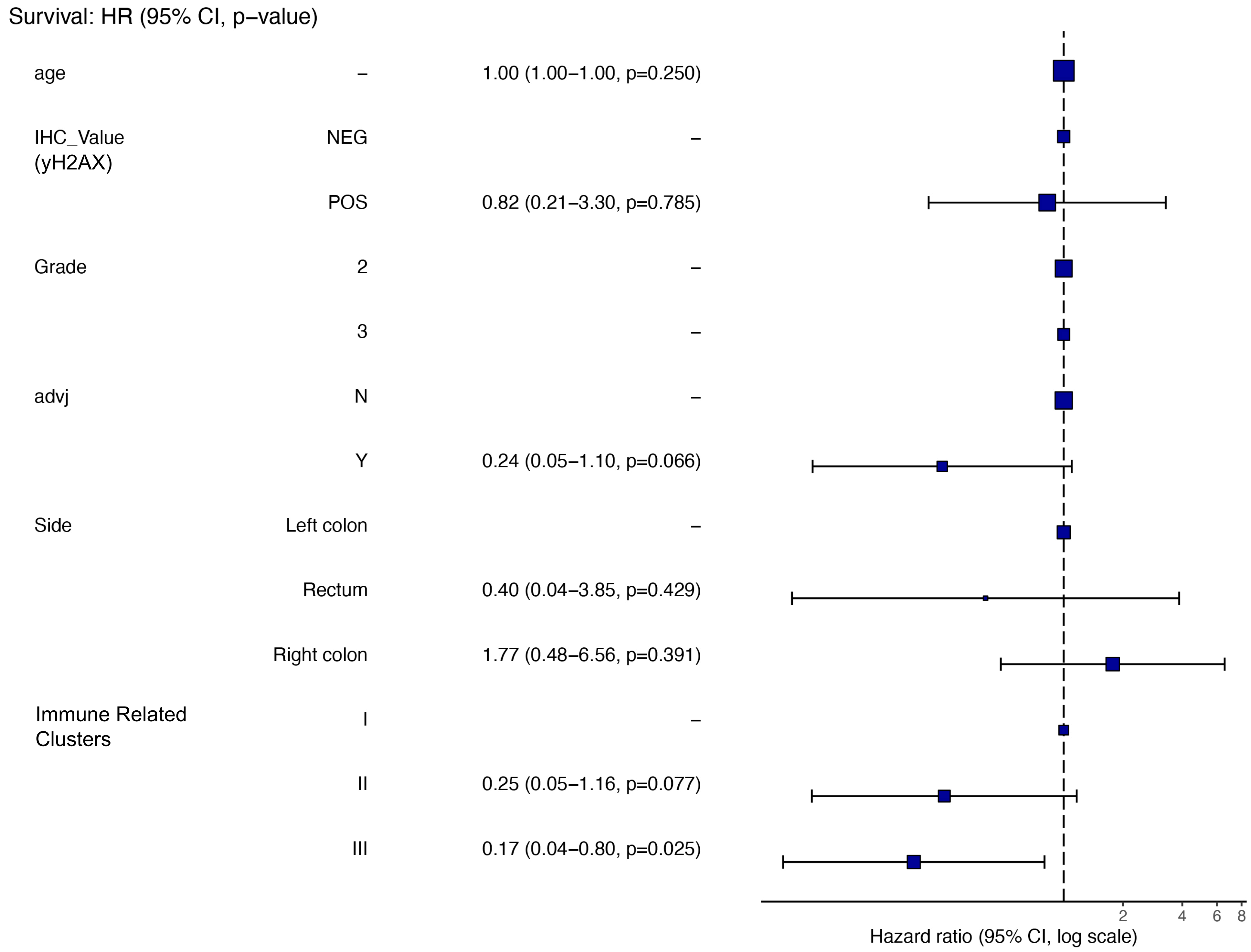
Survival: HR (95% CI, p−value)
age
−
1.00 (1.00−1.00, p=0.250)
IHC_Value (yH2AX)
NEG
−
POS
0.82 (0.21−3.30, p=0.785)
Grade
2
−
3
−
advj
N
−
Y
0.24 (0.05−1.10, p=0.066)
Side
Left colon
−
Rectum
0.40 (0.04−3.85, p=0.429)
Right colon
1.77 (0.48−6.56, p=0.391)
Immune Related Clusters
I
−
II
0.25 (0.05−1.16, p=0.077)
III
0.17 (0.04−0.80, p=0.025)
2
4
6
8
Hazard ratio (95% CI, log scale)
