# Supplementary material for: Degrees of H2AX phosphorylation correlate with unique features of the intratumoral immune microenvironment in colorectal carcinomas
Source: Oncologist. 2026 Mar 30;31(5):oyag116. doi: 10.1093/oncolo/oyag116 (PMC13071407; doi:10.1093/oncolo/oyag116)
Supplement: oyag116_Supplementary_Data [file oyag116_supplementary_data.zip › Supplementary Figure 6_rev1.pdf]

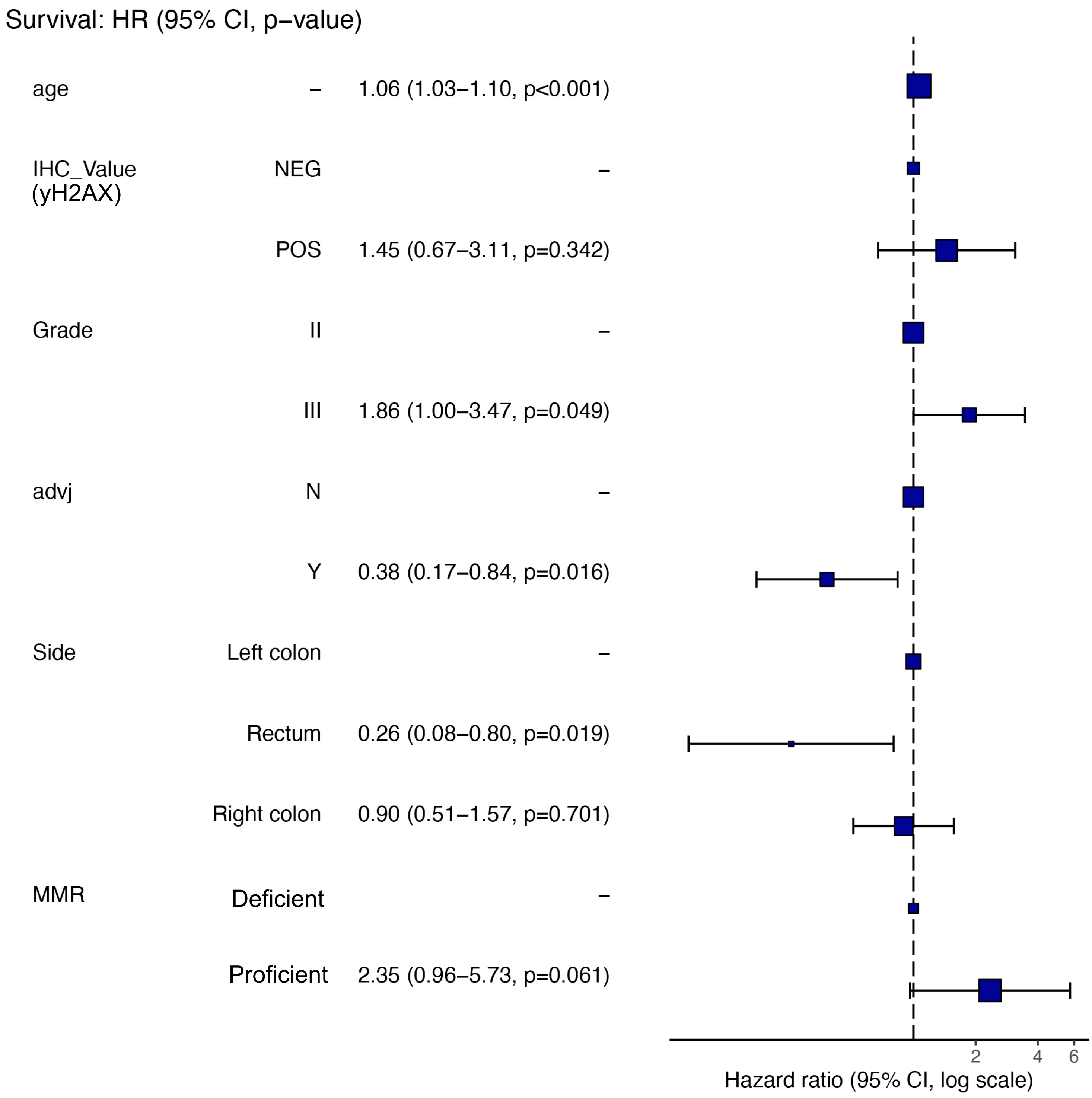

Survival: HR (95% CI, p−value)
age
−
1.06 (1.03−1.10, p<0.001)
IHC_Value (yH2AX)
NEG
−
POS
1.45 (0.67−3.11, p=0.342)
Grade
II
−
III
1.86 (1.00−3.47, p=0.049)
advj
N
−
Y
0.38 (0.17−0.84, p=0.016)
Side
Left colon
−
Rectum
0.26 (0.08−0.80, p=0.019)
Right colon
0.90 (0.51−1.57, p=0.701)
MMR
Deficient
−
Proficient
2.35 (0.96−5.73, p=0.061)
2
4
6
Hazard ratio (95% CI, log scale)
