# Supplementary material for: Degrees of H2AX phosphorylation correlate with unique features of the intratumoral immune microenvironment in colorectal carcinomas
Source: Oncologist. 2026 Mar 30;31(5):oyag116. doi: 10.1093/oncolo/oyag116 (PMC13071407; doi:10.1093/oncolo/oyag116)
Supplement: oyag116_Supplementary_Data [file oyag116_supplementary_data.zip › Supplemental Figure Legends_rev1.docx]

**Supplemental figure 1. A.** Boxplot of *CD3D* RNA expression in yH2AX negative and positive CRCs**. B.** Boxplot of *CD3E* RNA expression in yH2AX negative and positive CRCs **C.** Boxplot of *CD3G* RNA expression in yH2AX negative and positive CRCs.

**Supplemental figure 2.A** *K*-mean clustering of raw immune cell count in the whole CRC cohort. **B.** Silhouette plot for best *k* identification. **C.** Distribution of MMR-based group within the raw immune clusters. **D.** Distribution of yH2Ax value-based group within the raw immune clusters in the whole cohort. **E** *K*-mean clustering of raw immune cell count in the MMR proficient, CRC cohort. **F.** Distribution of yH2Ax value-based group within the raw immune clusters in the MMR proficient cohort.

**Supplemental figure 3.A** *K*-mean clustering of relative immune cell count in the whole CRC cohort. **B.** Silhouette plot for best *k* identification for the whole cohort. **C.** Distribution of yH2Ax value-based group within the raw immune clusters. **D.** *K*-mean clustering of relative immune cell count in the MMR deficient, CRC cohort. **E.** Silhouette plot for best *k* identification for the MMR deficient cohort. **F.** Distribution of yH2Ax value-based group within the relative immune clusters in the MMR deficient cohort.

**Supplemental figure 4. A-B-C.** Volcano plot of differentially expressed genes between the relative immune clusters of the MMR proficient cohort.

**Supplemental figure 5.** Heatmap of the DNA damage repair (DDR) related genes for MMR proficient patients belonging to relative immune cluster 1 and 2.

**Supplemental figure 6.** Forest plot reporting the hazard ratio of overall survival for the main clinico and pathological features in the whole cohort of CRCs, comprising also the yH2AX value-based groups.

**Supplemental figure 7.** Overall survival Kaplan-Meier curves with HR and p-value for the yH2AX value-based groups.

**Supplemental figure 8.** Forest plot reporting the hazard ratio of overall survival for the main clinico and pathological features in the MMR proficient cohort analyzed for the gene expression profiling, comprising also the yH2AX value-based and the immune related clusters.
