# Supplementary figures and images for: Degrees of H2AX phosphorylation correlate with unique features of the intratumoral immune microenvironment in colorectal carcinomas
Source: Oncologist. 2026 Mar 30;31(5):oyag116. doi: 10.1093/oncolo/oyag116 (PMC13071407; doi:10.1093/oncolo/oyag116)

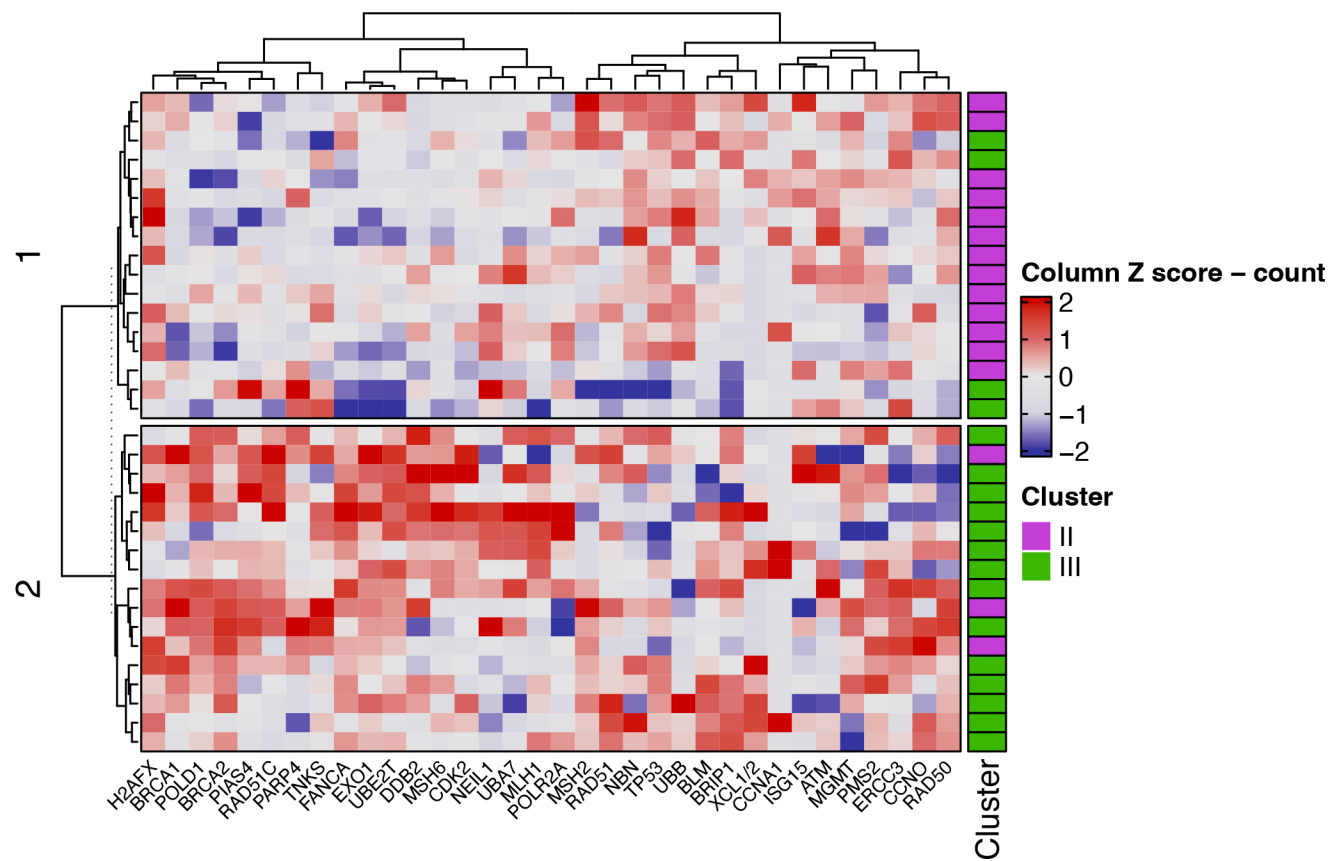

Supplement: oyag116_Supplementary_Data [file oyag116_supplementary_data.zip › Supplementary Figure 5_rev1.pdf]

yH2Ax    + NEG    + POS

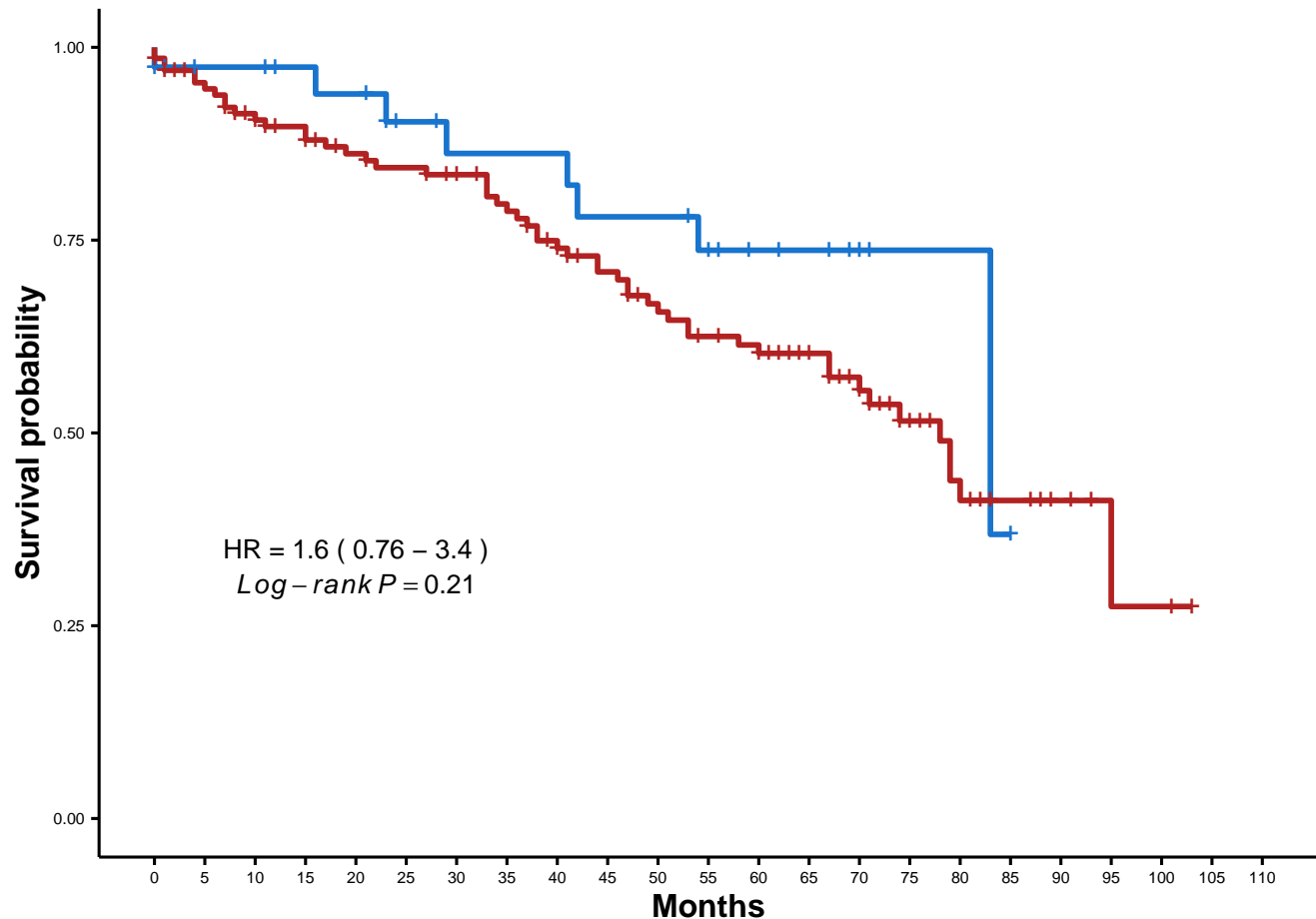

**Number at risk**

|            |     |     |     |     |    |    |    |    |    |    |    |    |    |    |    |    |    |    |   |   |   |   |   |
|------------|-----|-----|-----|-----|----|----|----|----|----|----|----|----|----|----|----|----|----|----|---|---|---|---|---|
| <b>NEG</b> | 39  | 30  | 30  | 28  | 27 | 23 | 21 | 21 | 21 | 19 | 19 | 17 | 12 | 10 | 5  | 2  | 2  | 1  | 0 | 0 | 0 | 0 | 0 |
| <b>POS</b> | 140 | 119 | 111 | 103 | 96 | 93 | 90 | 84 | 76 | 69 | 63 | 58 | 56 | 42 | 33 | 23 | 17 | 10 | 7 | 3 | 2 | 0 | 0 |

Supplement: oyag116_Supplementary_Data [file oyag116_supplementary_data.zip › Supplementary Figure 7_rev1.pdf]

**A**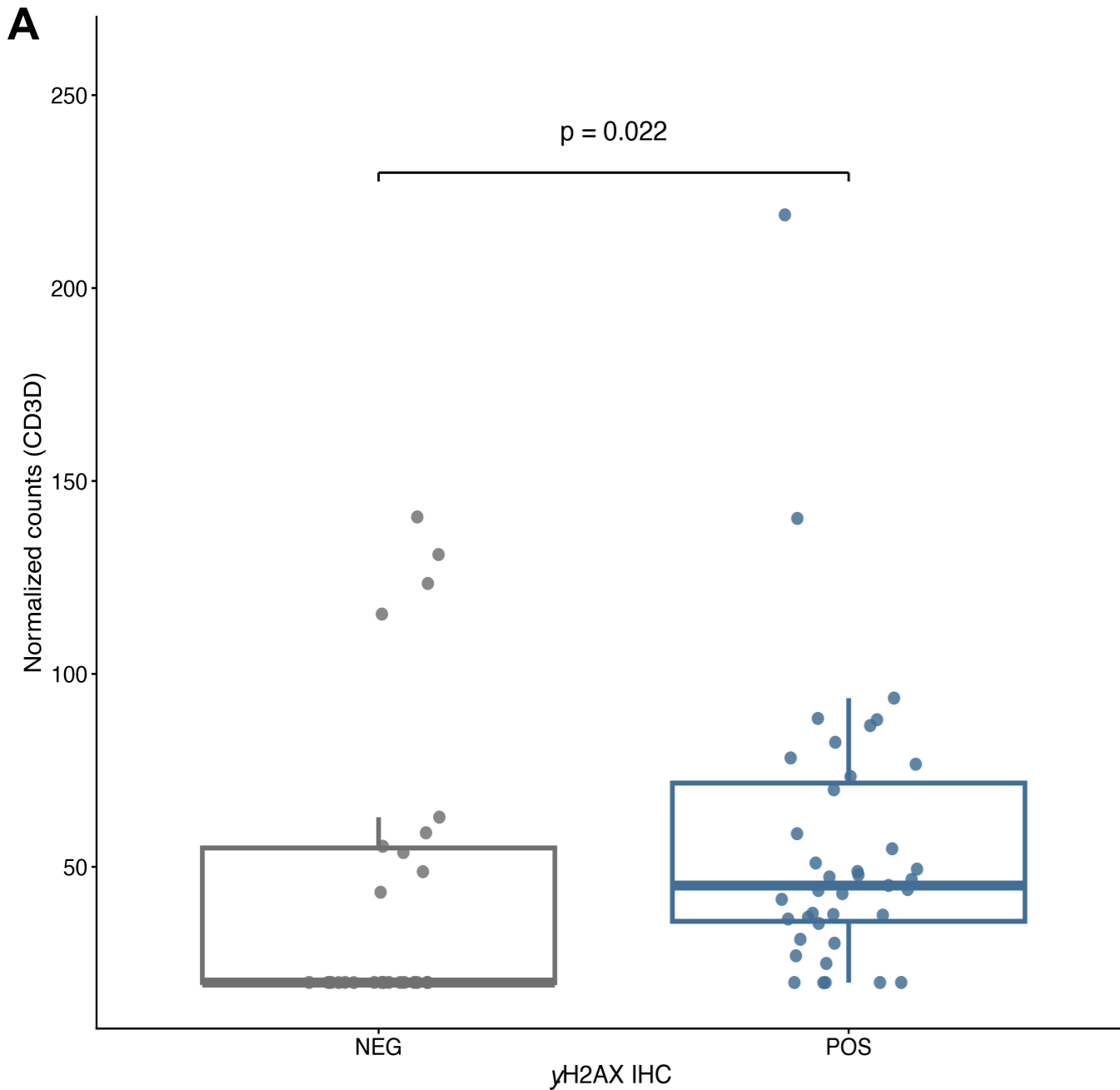**B**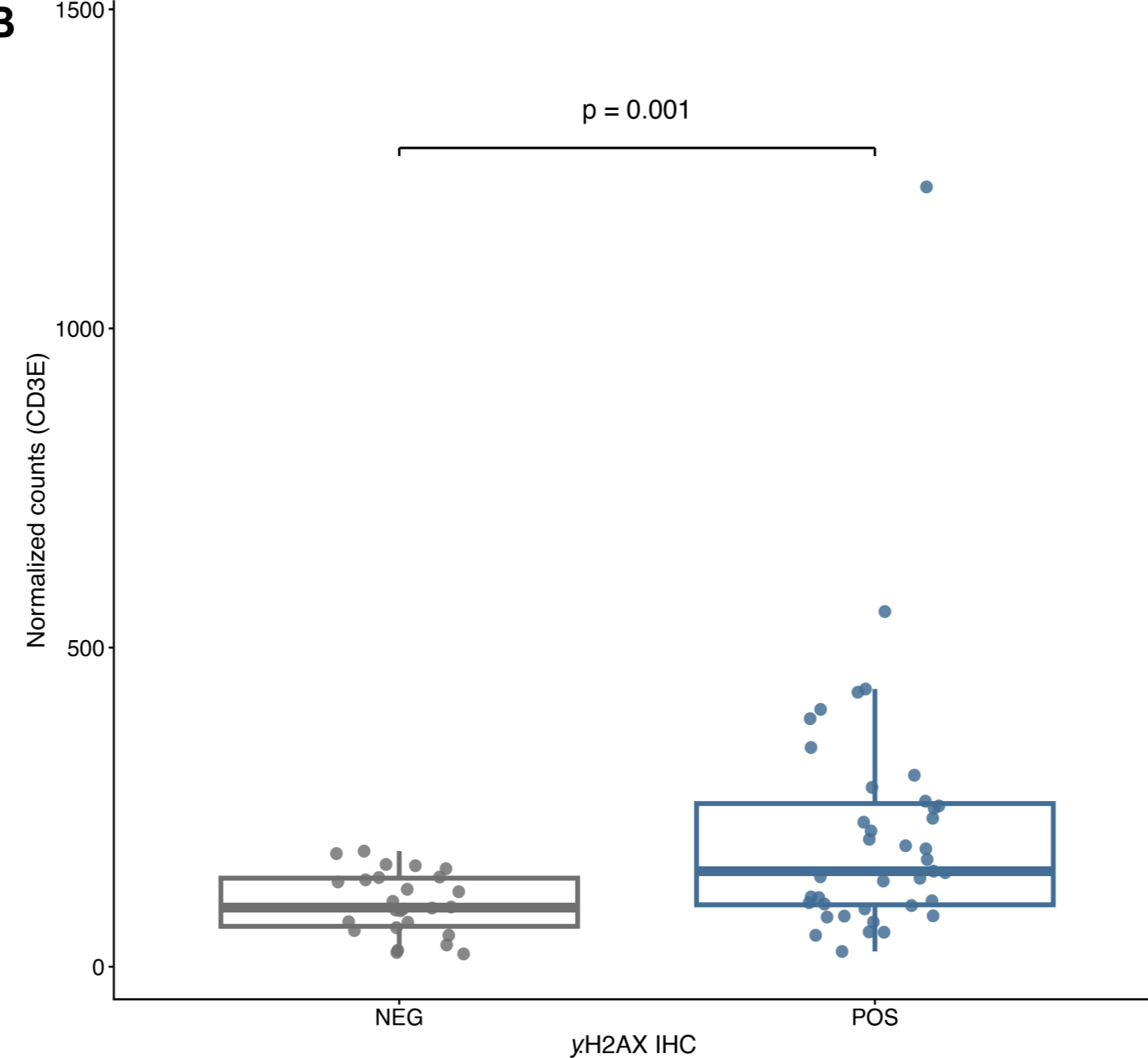**C**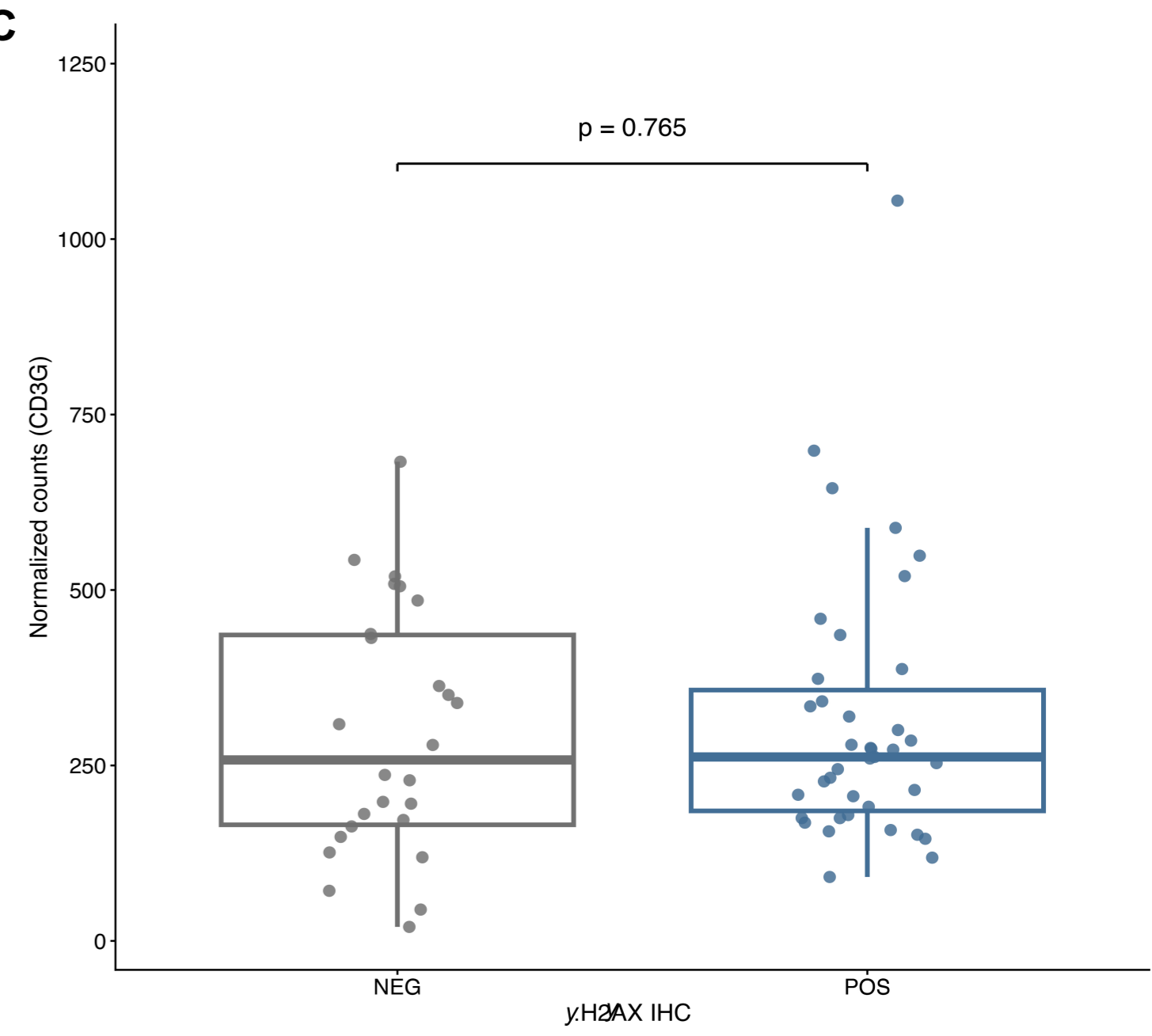

Supplement: oyag116_Supplementary_Data [file oyag116_supplementary_data.zip › Supplementary Figure 1_rev1.pdf]
